# Fever Associated with Gastrointestinal Shigellosis Unmasks Probable Brugada Syndrome

**DOI:** 10.1155/2009/492031

**Published:** 2009-12-24

**Authors:** John N. Makaryus, Jennifer Verbsky, Scott Schwartz, David Slotwiner

**Affiliations:** Department of Cardiology, North Shore-Long Island Jewish Health System, Manhasset, 270-05 76th Ave, New Hyde Park, NY 11040, USA

## Abstract

Since it was first described approximately 15 years ago, the Brugada Syndrome has spurred a significant quantity of interest in its underlying mechanism and physiology. The Brugada electrocardiographic pattern is characterized by right bundle branch block morphology and ST segment elevations in the right precordial leads with an absence of identifiable underlying structural heart disease. The syndrome is clinically significant since these patients are at a higher risk of developing malignant ventricular arrhythmias. One of the mechanisms behind the disorder involves mutations in specific myocardial sodium channels. Furthermore, these electrocardiographic changes appear to be temperature dependent. We present the case of a 35-year-old male who presented with intestinal Shigellosis and was also found to have Brugada-type electrocardiographic changes on ECG. The electrocardiographic changes that were present when the patient was admitted and febrile resolved following antibiotic therapy and defervescence.

## 1. Case Presentation

A 37-year-old man with a history of mitral valve prolapse presented to the emergency department (ED) with a two-day history of fevers (up to 105° Fahrenheit (F) at home) and twelve episodes of grossly bloody diarrhea, which began approximately two hours after eating take-out Chinese food. The patient also reported dizziness and lightheadedness upon standing, likely secondary to dehydration from significant quantities of diarrhea.

In the ED, the patient was febrile with a temperature of 103.5°F, tachycardic with a heart rate of 114 beats per minute, and orthostatic, but was otherwise hemodynamically stable. Laboratory studies were unremarkable. In addition to aggressive intravenous (IV) hydration, the patient was started on IV ciprofloxacin and metronidazole for the empiric treatment of bacterial gastroenteritis. Although blood and urine cultures were negative, the patient's stool culture found moderate S*higella sonnei*. The patient's diarrhea gradually resolved following initiation of IV antibiotics and he was subsequently discharged on oral ciprofloxacin to complete a seven-day course of therapy.

**Figure 1:**
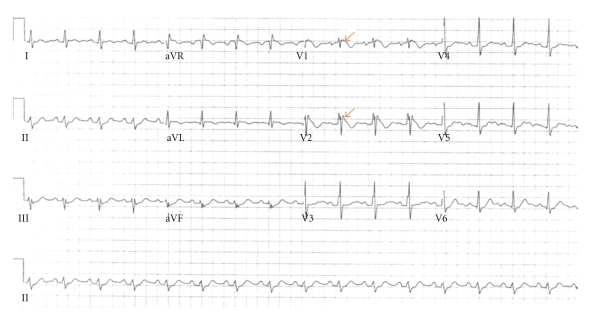
ECG upon admission demonstrating ST-segment elevations in leads V1 and V2 and incomplete right-bundle-branch block pattern, findings consistent with Brugada type 1 pattern. The patient was febrile to 103°F when this ECG was performed.

**Figure 2:**
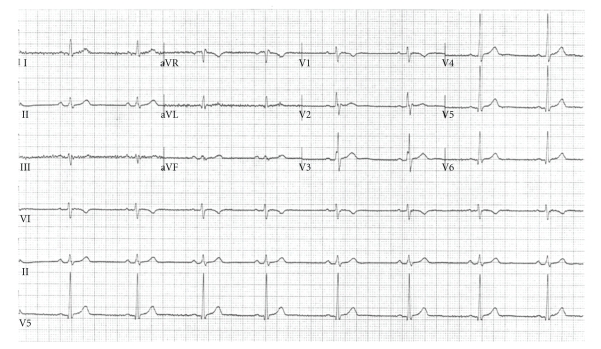
The patient's ECG on hospital day 2 after he had been treated with intravenous antibiotics and had defervesced. The ECG demonstrates minor ST changes but resolution of the findings seen up on admission.

On admission, a routine electrocardiogram (Figure 1) incidentally demonstrated an incomplete right bundle branch block along with 1.5 mm ST segment elevations with descent into inverted T waves in leads V1 and V2. On hospital day 1, a repeat ECG demonstrated similar findings. A subsequent ECG on hospital day 2 (Figure 2), when the patient was afebrile, demonstrated resolution of the previously seen electrocardiographic findings and restoration of a regular sinus bradycardia. Detailed questioning of the patient (and his mother) revealed that there was no reported family history of sudden cardiac death or known arrhythmias. The patient's father did have coronary artery disease and had undergone percutaneous coronary intervention at the age of 67. Otherwise, neither the patient nor his family members had ever heard of the term “Brugada” and were not aware of any other significant family history of cardiac disease. The patient himself had reportedly had an episode where he “passed out” approximately eight years prior to presentation and had a reportedly normal electrocardiogram and an echocardiogram which demonstrated mitral valve prolapse but was otherwise unremarkable. He stated that his doctor at the time told him that he was likely dehydrated. The patient otherwise denied any chronic chest pain, shortness-of-breath, palpitations, or any limitation of his exercise tolerance. Given the lack of cardiovascular symptoms during the current episode and resolution of previously seen ECG findings after defervescence, the patient was discharged. He followed up with an electrophysiologist following discharge and had a repeat ECG which was unremarkable. He opted not to pursue any further testing but will follow regularly with his outpatient physician for followup of his abnormal ECG.

## 2. Discussion

The patient clearly suffered from gastrointestinal shigellosis given the presence of crampy abdominal pain, bloody diarrhea, and stool studies that positively identified the organism. The microorganism was sensitive to ciprofloxacin and the patient showed signs of clinical improvement relatively quickly after initiation of therapy. The electrocardiographic findings, however, quickly became the focus of his care.

The electrocardiographic findings present on the first ECG are consistent with the Brugada pattern. The Brugada syndrome is divided into three types based upon the morphology of the electrocardiographic changes. Type 1 is characterized by coved ST-segment elevations in the right precordial leads followed by T-wave inversions. The ST elevations in type 2 Brugada syndrome are usually described as having a “saddleback” appearance with a high takeoff ST-segment elevation followed by a trough with a less pronounced ST elevation and a subsequent biphasic or positive T wave. The third and final Brugada pattern has ST elevations with either a coved or saddleback appearance with ST-segment elevations of 1 mm [1]. 

The ECG in the case presented above demonstrates type 1 Brugada morphology with coved ST-segment elevations in leads V1-V2 followed by T-wave inversions. Furthermore, in this case, the diagnosis is clinched by the clinical circumstances in which the electrocardiographic findings were appreciated. The Brugada syndrome is defined as RBBB pattern (really J-point elevation) with ST-segment elevations in leads V1-V3 and syncope, documented ventricular fibrillation, sudden cardiac death (SCD) in a patient with a structurally normal heart, or a family history of sudden cardiac death in a family member less than 45 years of age [1]. In addition, the electrocardiographic findings associated with Brugada syndrome can often be elicited by sodium channel blockers, vagotonic agents, or by a febrile illness, as was most likely the case here [2–5]. 

The etiology of the Brugada syndrome has been investigated extensively over the past fifteen years. Data suggest a likely genetic predisposition stemming from mutations in the genes encoding specific subunits of myocardial sodium channels [6]. Approximately 25% of all Brugada patients have been shown to have a mutation in the cardiac sodium channel gene *SCN5A* located on chromosome 3. Other genetic mutations, including alterations of the *β*-subunit of cardiac sodium channels, may also be associated with a greater risk of arrhythmogenicity and Brugada syndrome. Anything that alters inflow or outflow through these sodium channels, including elevated core body temperature, may unmask the electrocardiographic findings associated with the Brugada syndrome [3, 5, 7]. As a result, in some patients, the Brugada syndrome may only be discovered when the core body temperature is elevated. While individuals with febrile illness may be predisposed to syncope as a result of potential orthostatic hypotension or vasovagal syncope which are far more common than arrhythmogenic causes of syncope, physicians should be aware of the association between fever and the Brugada syndrome. However, the prognostic value of Brugada morphology ECG changes observed only during a febrile illness remains unknown.

The Brugada syndrome is estimated to account for almost five percent of all sudden cardiac deaths and about a fifth of all deaths in patients with no evidence of structural cardiac abnormalities [8]. As a result, careful electrocardiographic analysis, even in individuals not suspected of having cardiovascular disease, is essential. The only proven effective treatment option for patients with the Brugada syndrome is the implantable cardioverter-defibrillator (ICD) [9, 10]. There are several specific indications for ICD implantation, including the presence of type 1 Brugada electrocardiographic pattern in the presence of symptoms (such as syncope, seizure, or nocturnal agonal respiration). Asymptomatic patients usually undergo electrophysiology studies (EPS) prior to ICD implantation. Even if asymptomatic, patients with the Brugada-type electrocardiographic pattern should be further evaluated. The utility of EPS for risk stratification has been debated, and the optimal means of identifying high-risk patients remains uncertain.
